# Cilostazol-induced acute tubulointerstitial nephritis accompanied by IgA nephropathy: a case report

**DOI:** 10.1186/s12882-018-0854-0

**Published:** 2018-03-05

**Authors:** Hisato Shima, Manabu Tashiro, Satoshi Yamada, Motokazu Matsuura, Kazuyoshi Okada, Toshio Doi, Jun Minakuchi, Shu Kawashima

**Affiliations:** 1 Department of Kidney Disease, Kawashima Hospital, 1-39 Kitasakoichiban-cho, Tokushima, 770-0011 Japan; 2 Department of Nephrology, Tokushima University Graduate School, 3-18-15 Kuramoto-cho, Tokushima, 770-8503 Japan

**Keywords:** Cilostazol, Acute tubulointerstitial nephritis, Acute kidney injury, IgA nephropathy, Urine beta2-microglobulin, Gallium-67 scintigraphy

## Abstract

**Background:**

Cilostazol is an antiplatelet drug that is widely prescribed for the prevention of secondary stroke. Adverse reactions to cilostazol include headaches, palpitations, and diarrhea. Little is known about the nephrotoxicity of cilostazol, such as acute kidney injury. We report a biopsy-proven case of diffuse tubulointerstitial nephritis induced by cilostazol.

**Case presentation:**

A 69-year-old woman prescribed cilostazol was hospitalized for acute kidney injury. On admission, her renal function deteriorated, with an increased serum creatinine level. Urinalysis showed hematuria, proteinuria, and hyper-beta2-microglobulinuria. A renal biopsy revealed diffuse tubulointerstitial nephritis associated with IgA nephropathy, and gallium-67 scintigraphy showed uptake in the bilateral kidneys. A drug lymphocyte stimulation test for cilostazol was positive, and the patient was diagnosed with cilostazol-induced acute tubulointerstitial nephritis. Despite discontinuation of cilostazol, her renal function rapidly worsened and steroid pulse therapy was initiated, followed by oral high-dose glucocorticoid therapy. After steroid treatment, her serum creatinine level normalized in parallel with urine beta2-microglobulin.

**Conclusion:**

Cilostazol can induce acute tubulointerstitial nephritis.

## Background

Cilostazol is a phosphodiesterase type III inhibitor and an antiplatelet drug for recurrent stroke prevention [1]. Reported side effects include headaches, palpitations, and diarrhea. However, nephrotoxicity has rarely been reported [2]. In many cases, doctors freely prescribe cilostazol without considering its potential nephrotoxicity. Acute tubulointerstitial nephritis (ATIN) is a major cause of acute renal dysfunction, with the main causes being drugs, infections, and autoimmune diseases [3]. Among drugs that can cause the disease, antibiotics and nonsteroidal anti-inflammatory drugs are common [4]. Here, we report a case of cilostazol-induced ATIN accompanied by immunoglobulin A (IgA) nephropathy. To the best of our knowledge, this is the first case report of cilostazol-induced ATIN confirmed by a renal biopsy. Although renal function improved with steroid treatment, interstitial nephritis relapsed when the dose was reduced from 25 mg/day to 20 mg/day. Generally, high-dose steroids such as 40–60 mg daily can be tapered quickly, because our bodies are oversaturated with steroids. The best speed at which to taper the steroid dose is usually difficult to determine at a lower dose, because the possibility of recurrence and appearance of many symptoms might be increased. The speed at which to taper the steroid dose appeared to be an important factor, even at a higher dose such as 40 mg per day.

## Case presentation

**Fig. 1:**
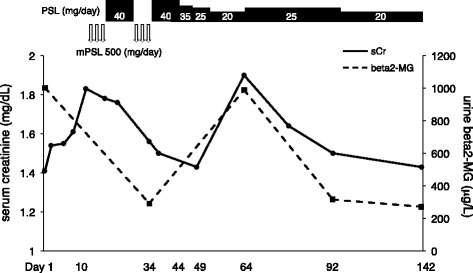
Serum creatinine and urine beta2-microglobulin. sCr, serum creatinine; MG, microglobulin; PSL, prednisolone; mPSL, methylprednisolone

**Fig. 2:**
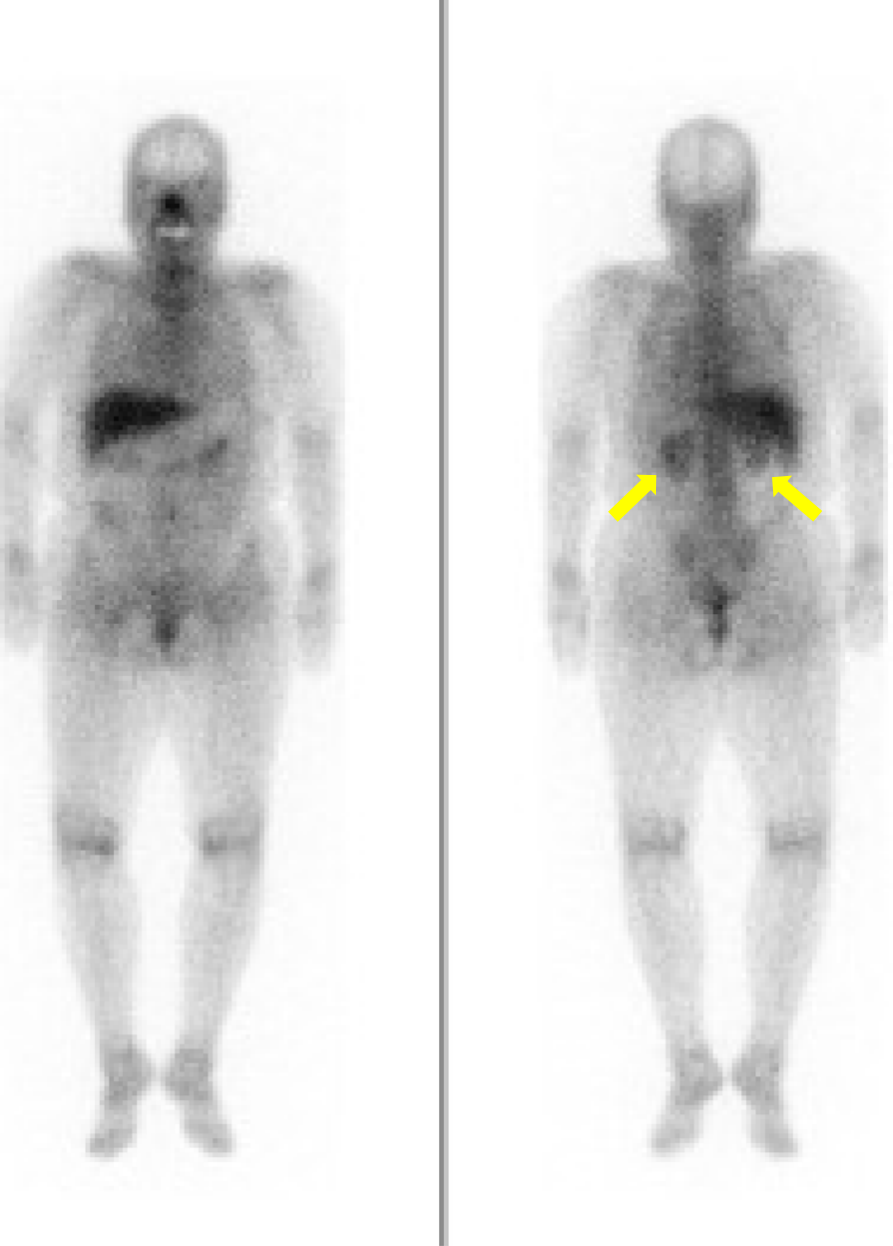
Gallium-67 scintigraphy. Gallium-67 scintigraphy revealed uptake in both kidneys

**Fig. 3:**
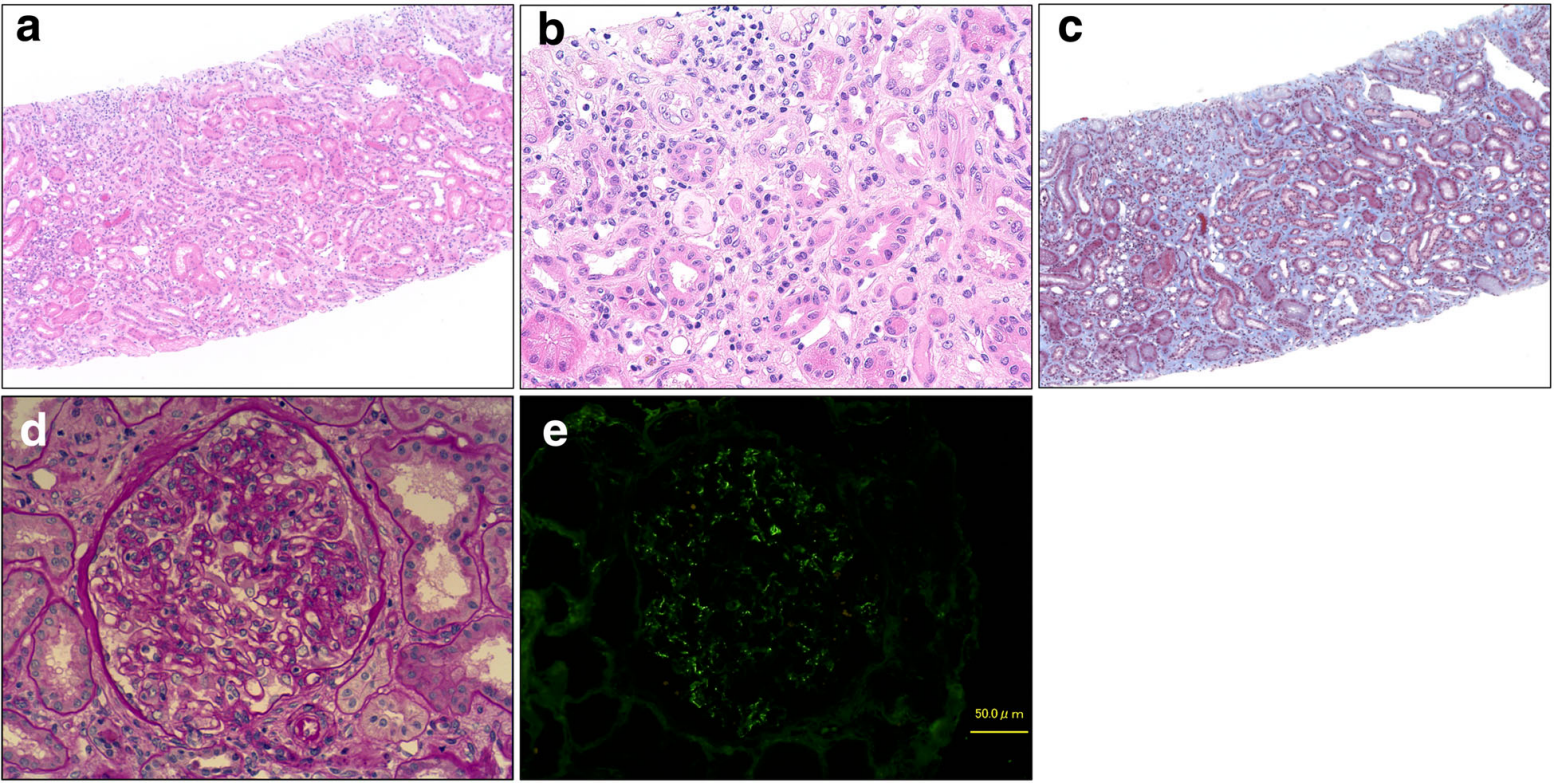
Renal biopsy specimen. **a b** Hematoxylin and eosin staining (**a** 100× magnification; **b** 400× magnification); **c** Masson trichrome staining (100× magnification); **d** Periodic acid–Schiff staining (400× magnification); **e** Immunofluorescence microscopy showing granular IgA mesangial deposits. IgA, immunoglobulin A

A 69-year-old woman (height, 148.7 cm; weight, 59.3 kg) was admitted to our hospital because of acute kidney injury. She had a history of right corona radiata infarct and had been taking cilostazol (50 mg) twice daily for to prevent recurrent cerebral infarction; cilostazol had been prescribed at another hospital 13 months before admission. She was not taking other medications and had no history of allergies. Her baseline serum creatinine (sCr) level was 0.85 mg/dL (normal, < 0.82 mg/dL) until 6 months before admission. On admission, her body temperature was 36.5 °C, blood pressure was 126/78 mmHg, and heart rate was 82/min. Blood tests revealed a creatinine level of 1.41 mg/dL. Urinalysis showed microscopic hematuria (urine red blood cells, > 100 per high-power field; urine white blood cells, 0–2 per high-power field), proteinuria (2.23 g/gCr; normal, < 0.15 g/gCr), and hyper-beta2-microglobulinuria (1002 μg/L; normal, < 289 μg/L). The serum IgE level was normal (80 U/mL). She had not had signs of headaches, palpitations, diarrhea, rash, flank pain, arthralgia, eosinophilia, or macroscopic hematuria. She did not present with glucosuria, nephrogenic diabetes insipidus, polyuria, or nocturia. The patient’s clinical course is shown in Fig. 1. A renal ultrasound showed that the kidneys were of normal size (right, 93 mm × 44 mm; left, 92 mm × 43 mm) with no dilation of the urinary tract, renal pelvis, or calyces. The corticomedullary junction was obscure and there were no kidney stones. The resistive index at the renal interlobular artery was normal (right, 0.62; left, 0.66). Gallium-67 scintigraphy revealed uptake in the bilateral kidneys (Fig. 2). A renal biopsy was performed after stopping oral cilostazol and revealed diffuse lymphocyte infiltration in the interstitium accompanied by mild interstitial fibrosis and tubular atrophy (Fig. 3a, b and c). Additionally, sparse acute tubular necrosis and hematic cylinder were recognized in a tubulointerstitial lesion (Fig. 3a and c). Focal segmental mesangial and intracapillary hypercellularity were observed (Fig. 3d). Crescents (one cellular and one fibrocellular) were observed in two of 13 glomeruli. There were no granulomas. Immunofluorescence staining showed a granular pattern for IgA (Fig. 3e), IgM, and C3 in the mesangium and was negative for IgG, IgG4, C1q, C4, and fibrinogen. A diagnosis of diffuse tubulointerstitial nephritis accompanied by IgA nephropathy was made. We suspected that the patient had delayed drug-induced hypersensitivity reactions, so a drug lymphocyte stimulation test (DLST) for cilostazol was performed. The ^3^H–thymidine uptake in peripheral blood mononuclear cells stimulated with the suspect drugs was evaluated [5]. The stimulation index (SI) is defined as the value of ^3^H–thymidine uptake with antigen/without antigen [5]. In our patient, the DLST result for cilostazol was positive (SI, 200%; a positive result is > 180%). Test results for p-anti-neutrophil cytoplasmic antibody, c-anti-neutrophil cytoplasmic antibody, anti-glomerular basement membrane antibody, anti-nuclear antibody, anti-Sjögren’s syndrome A, and anti-Sjögren’s syndrome B antibody were negative. Test results for hepatitis B, hepatitis C, human immunodeficiency virus, cytomegalovirus, and Epstein–Barr virus were negative. No abnormalities were observed on ophthalmological examination. Her serum angiotensin-converting enzyme level was normal. Therefore, we excluded other causes of ATIN, such as systemic lupus erythematosus, Sjögren’s syndrome, IgG4-related diseases, certain acute viral infections, tubulointerstitial nephritis, uveitis syndrome, and sarcoidosis [4]. The patient was diagnosed with cilostazol-induced diffuse tubulointerstitial nephritis accompanied by IgA nephropathy; the IgA nephropathy might have existed before the administration of cilostazol.

We administered glucocorticoid pulse therapy with methylprednisolone (500 mg/day) for 3 days and then reduced the prednisolone dose to 40 mg/day for 2 weeks. After two courses of this regimen, we reduced the dose of prednisolone to 35 mg/day on day 44 and then to 25 mg/day. As shown in Fig. 1, urinary beta2-microglobulin on day 34, and sCr and urinalysis on day 49 improved (urine beta2-microglobulin, 291 μg/L; sCr, 1.43 mg/dL; urine red blood cells, 11–30 per high-power field). The patient was discharged on 20 mg/day prednisolone. However, sCr, urinalysis, and urinary beta2-microglobulin levels increased (sCr, 1.90 mg/dL; urine red blood cells, > 100 per high-power field; urine beta2-microglobulin, 989 μg/L) by day 64, indicating recurrence of interstitial nephritis (Fig. 1). The dose of prednisolone was increased to 25 mg/day for 4 weeks and then reduced to 20 mg/day without recurrence.

## Discussion

This case highlights two important clinical observations. First, cilostazol appeared to be responsible for the patient’s serious side effects, including acute kidney injury, because cilostazol was the only drug administered when she was admitted. From the results of the renal biopsy, gallium-67 scintigraphy, and the DLST, we diagnosed ATIN induced by cilostazol. ATIN is reported to have three classical symptoms: fever, rash, and eosinophilia [4]. However, as seen in this case, these symptoms were not present, and are reported to occur in only 10% of patients with ATIN [6]. According to the Gell and Coombs classification, drug-induced ATIN is a type IV delayed hypersensitivity reaction, which is mediated by drug-reactive T lymphocytes [7]. Although drug-induced ATIN is usually temporally related to drug therapy, the duration of therapy before development of ATIN is different for different medicines. For example, ATIN typically develops at 7–10 days after starting a medication [3]. However, for nonsteroidal anti-inflammatory drugs, the interval between starting a medication and the development of ATIN is reported to be longer (6–18 months) [3]. In this patient, the serum creatinine (sCr) level was 0.85 mg/dL at 7 months and it increased to 1.41 mg/dL at 13 months after starting cilostazol therapy. It is difficult to clarify when kidney injury started, because the patient did not have a blood test for 6 months. ATIN might have developed at 7–13 months after starting cilostazol therapy. There are few reports of acute kidney injury caused by cilostazol. Nomoto et al. reported acute renal failure as an adverse drug reaction to cilostazol [2]. However, the mechanism was not determined in that study because the researchers did not perform a renal biopsy, gallium-67 scintigraphy, or DLST. To our knowledge, this is the first case in which cilostazol has been shown to induce tubulointerstitial nephritis.

Second, this case suggests that gradual tapering of steroids, even at high doses, is important. Early steroid treatment is useful after a diagnosis of drug-induced ATIN [8]. Despite high-dose oral steroids (1 mg/kg/day or 60 mg/day) being common [9], the appropriate dosage and duration of steroid treatment remains uncertain. Pusey et al. reported that the renal function of all ATIN patients treated intravenously with high-dose methylprednisolone (500 mg/day or 1000 mg/day) returned to normal [10]. In the current case, diffuse lymphocyte infiltration was severe and accompanied by IgA nephropathy, so we conducted methylprednisolone pulse therapy (500 mg/day). A previous publication reported that three courses of high-dose methylprednisolone (500 mg/day) were used to treat a patient with ATIN, whose renal biopsy specimens revealed severe inflammatory cell infiltration [11]. Because the initial dose was high, we administered 40 mg/day after glucocorticoid pulse therapy.

During treatment, the patient experienced steroid-related side effects, including diabetes, insomnia, and moon face, and as her serum IgG level decreased, she was at increased risk for infection. These factors were a cause for concern and we tapered the steroid treatment. Interstitial nephritis relapsed with a dose reduction from 25 mg/day to 20 mg/day. Although high-dose steroids can be tapered quickly [12], it is suggested that tapering should be as slow as possible even at a high dose to avoid a flare up.

Urinary beta2-microglobulin is reported to be a useful indicator of renal tubulointerstitial damage [13]. The patient’s worsening sCr level was thought to be because of ATIN, which is consistent with the increase in urine beta2-microglobulin. Gallium-67 scintigraphy is useful for differentiating ATIN from acute tubular necrosis [3, 14]. If a patient has glomerulonephritis, its interpretation is not conclusive but supportive of tubulointerstitial changes in the kidney. Hematuria is a key symptom in IgA nephropathy [15]. In the current patient, hematuria may have been a symptom of both ATIN and IgA nephropathy. According to the Oxford Classification of IgA nephropathy [16], this patient’s score is M1E1S1T1C1 (M1, glomeruli showing mesangial hypercellularity > 50%; E1, endocapillary hypercellularity present; S1, segmental glomerulosclerosis present; T1, tubular atrophy/interstitial fibrosis, 26–50%; C1, cellular or fibrocellular crescents, 0–25%). Therefore, IgA nephropathy for this patient was also active and might be responsive to steroid treatment. Hematic cylinder would also be a reason for elevated creatinine. Additionally, uromodulin might be present in the Bowman’s space and in some tubules, which suggests an obstructive cause. However, diffuse tubulointerstitial nephritis independent of glomerular damage (Fig. 3a, b and c) is thought to be induced by cilostazol. ATIN concomitant with IgA nephropathy induced renal dysfunction. After steroid treatment, sCr recovered in parallel with urine beta2-microglobulin.

## Conclusions

We report the rare case of a patient with cilostazol-induced ATIN accompanied by IgA nephropathy. Steroid therapy was successful but the tapering speed was important. Because all patients with ATIN have acute renal failure [4], attention should be paid to renal parameters such as sCr and urinalysis after administration of cilostazol.

## Abbreviations

ATIN: Acute tubulointerstitial nephritis
DLST: Drug lymphocyte stimulation test
Ig: Immunoglobulin
sCr: Serum creatinine
